# Comparison of Glucose and Lipid Metabolic Gene Expressions between Fat and Lean Lines of Rainbow Trout after a Glucose Load

**DOI:** 10.1371/journal.pone.0105548

**Published:** 2014-08-20

**Authors:** Junyan Jin, Françoise Médale, Biju Sam Kamalam, Peyo Aguirre, Vincent Véron, Stéphane Panserat

**Affiliations:** INRA, UR 1067 Nutrition Metabolism Aquaculture, Institut National de la Recherche Agronomique, Saint-Pée-sur-Nivelle, France; Universitat de Barcelona, Spain

## Abstract

Two experimental rainbow trout lines developed through divergent selection for low (Lean ‘L’ line) or high (Fat ‘F’ line) muscle fat content were used as models to study the genetic determinism of fat depots. Previous nutritional studies suggested that the F line had a better capability to use glucose than the L line during feeding trials. Based on that, we put forward the hypothesis that F line has a greater metabolic ability to clear a glucose load effectively, compared to L line. In order to test this hypothesis, 250 mg/kg glucose was intraperitoneally injected to the two rainbow trout lines fasted for 48 h. Hyperglycemia was observed after glucose treatment in both lines without affecting the phosphorylation of AMPK (cellular energy sensor) and Akt-TOR (insulin signaling) components. Liver glucokinase and glucose-6-phosphate dehydrogenase expression levels were increased by glucose, whereas mRNA levels of β-oxidation enzymes (CPT1a, CPT1b, HOAD and ACO) were down-regulated in the white skeletal muscle of both lines. Regarding the genotype effect, concordant with normoglycemia at 12 h after glucose treatment, higher muscle glycogen was found in F line compared to L line which exhibited hyperglycemia. Moreover, mRNA levels of hepatic glycolytic enzymes (GK, 6PFK and PK), gluconeogenic enzyme PEPCK and muscle fatty acid oxidation enzymes (CPT1a, CPT1b and HOAD) were concurrently higher in the F line. Overall, these findings suggest that F line may have a better ability to maintain glucose homeostasis than L line.

## Introduction

Compared to mammals that have the ability to deal rapidly with a glucose load or a diet rich in carbohydrates, carnivorous teleost are generally recognized as poor users of glucose [1]–[4]. For instance, the glucose clearance time (recovery period back to baseline glycemia) after a glucose load was 12 h in European sea bass [5] and 24 h in rainbow trout [6], [7]. Despite the fact that almost all the enzymes involved in glucose metabolism have been characterized in fish, the regulation of glucose homeostasis in carnivorous fish does not follow mammalian patterns [4], [8]. In mammals, dietary carbohydrate intake significantly induces the expression of glycolytic enzymes and concurrently inhibits the expression of gluconeogenic enzymes [9], [10]. In carnivorous fish such as rainbow trout and gilthead sea bream, the expression of glucokinase was dramatically induced by a carbohydrate rich diet intake [11]–[13]. Accelerated glycolysis after a glucose load was similarly observed in red sea bream and yellowtail, however, concomitantly there was a small increase in gluconeogenic enzyme activity [14]. Impaired regulation of endogenous glucose synthesis by high glucose influx was also prominent in gilthead sea bream and rainbow trout [12], [15], [16]. Studies using deuterated water revealed that glucose appearance is almost entirely derived from gluconeogenesis, regardless of the nutritional status in European sea bass [17]. Thus, the lack of regulation of gluconeogenesis could be a reason behind the inefficient use of dietary carbohydrates in carnivorous fish such as rainbow trout [8], [18]. On the other hand, the storage of excess glucose as glycogen is effective in different species of fish, when fed a carbohydrate-rich diet [19], [20].

It is well known that glucose and fatty acid has a strong interaction, which can affect glucose utilization [21]. *De novo* lipogenesis plays a pivotal role in glucose homeostasis because glucose in excess can be converted into lipids. Many of the lipogenic enzymes are known to be induced by a high-carbohydrate diet in rats and mice [22]. As in mammals, lipogenic enzyme activities were up-regulated by dietary carbohydrate in coho salmon [23], [24] and *de novo* lipogenesis was linked to a decrease of hyperglycemia in rainbow trout after a glucose load [4]. But conflictingly, poor induction of lipogenesis was observed in rainbow trout fed a carbohydrate-rich diet [15] and *de novo* lipid synthesis from dietary starch was found to be minor in juvenile gilthead sea bream, using a stable isotope method [20]. In mammals, high carbohydrate availability reduces fatty acid oxidation via an increase in plasma insulin and decreased free fatty acid availability [25], [26]. On the contrary, in rainbow trout, regulation of enzymes involved in fatty acid oxidation was not dependent on the carbohydrate content of the diet [16], [27], [28]. Even conversely, glucose treatment was found to stimulate the rate of hepatic lipolysis by increasing the activity of hepatic lipase activity in rainbow trout [29].

Interestingly, differences were noted in glucose tolerance between Chinook salmon strains, which indicated that genotype may be an important determinant of glucose utilization capacity [30]. Recently, two experimental rainbow trout lines have been developed through divergent selection for low or high muscle fat content (designated L line and F line respectively), in order to study the genetic determinism of lipid depots [31]. Previous nutritional studies suggested that the F line had enhanced capability to use glucose in both liver and muscle, coupled with reduced hepatic fatty acid oxidation, as compared to the L line, at the 3rd generation of selection [32]. Further in the 4th generation, the F line showed a better ability of plasma glucose clearance through enhanced lipogenesis driven by an augmentation in TOR signaling pathway [33]. In the same generation, the F line also displayed a superior ability to store excess glucose due to enhanced hepatic lipogenic potential and higher liver glycogen content than the L line [16]. Taking all these information into account, we put forth the hypothesis that the F line has a better capability to maintain glucose homeostasis than L line after a glucose load. Since glucose tolerance tests (GTTs) are widely used to investigate the ability of the fish to utilize carbohydrates, by providing an indication of the potential use of high glucose loads [5], [34]–[36], we intraperitoneally administered glucose (250 mg/kg) into the two trout lines. Our objective was to investigate the regulation of glucose metabolism and lipid metabolism following glucose loading. We analyzed the plasma levels of metabolites such as glucose, triglycerides and free fatty acids at 3, 8 and 12 h post-injection. In liver and white muscle, we examined the phosphorylation status of certain components of insulin (Akt) and energy signaling (AMPK) pathway at 3 h post-injection. Importantly, as metabolic regulation by glucose occurs mainly at the transcriptional level [22], we assessed the mRNA levels of target genes involved in glucose transport, glycolysis, gluconeogenesis, lipogenesis and fatty acid β-oxidation, in liver and muscle, at the three post-injection time intervals.

## Materials and Methods

### Ethics Statement

The experiment was carried out in strict accordance with EU legal frameworks related to the protection of animals used for scientific purposes (Directive 2010/63/EU) and guidelines of the French legislation governing the ethical treatment of animals (Decree no. 2001-464, May 29th, 2001). It was approved by the Direction Departementale des Services Veterinaires (French veterinary services) to carry out animal experiments (INRA 2002–36, April 14th, 2002). The INRA experimental station is certified for animal services under the permit number A64.495.1 by the French veterinary services, which is the competent authority.

### Experimental design and sampling procedure

The study was conducted with two lines of rainbow trout (*Oncorhynchus mykiss*, Walbaum 1792), designated as Lean line (L) and Fat line (F), obtained after five generations of divergent selection for high or low muscle fat content using a nondestructive method (Distell Fish Fatmeter) as detailed by Quillet *et al.* [31]. Muscle fat content was found more than 3 times higher in the F line than in the L in 200 g-trout of this fifth generation [37]. Fish of L line (mean weight 237±47 g) and F line (mean weight 191±49 g) were maintained in re-circulatory aquaculture tanks with well-aerated water (water speed: 4 L/min, dissolved oxygen level: 10 mg/L) at a fairly constant temperature of 18°C and were fed a commercial diet (T-3P classic, Skretting, France) during the acclimatization period.

Fish were kept unfed for 48 h before the time of treatment in order to obtain fish with basal levels of plasma metabolites and an empty digestive tract. Fish were lightly anaesthetized with 0.05% (v/v) 2-phenoxyethanol and weighed. Then they received an intraperitoneal injection of glucose (250 mg/kg) or saline solution at 100 µl/100 g body mass, and were distributed into duplicate tanks per treatment. Four fish per tank (two tanks per condition) were euthanized by placing into a bath of excess 2-phenoxyethanol and immersing until death, then sampled at 3, 8 and 12 h after treatment (*n* = 8 per line at each sampling time). Blood was quickly removed from the caudal vein using syringes rinsed with an anticoagulant (0.04 g/ml potassium oxalate and 0.02 g/ml sodium fluoride) and then centrifuged (3000 *g*, 5 min). The recovered plasma was immediately frozen and kept at −20°C until analysis. Liver and a sample of dorso-ventral white muscle were dissected and cut in small pieces, immediately frozen in liquid nitrogen and then stored at −80°C pending analyses. All fish were sampled at the expected time ±5 min.

### Biochemical analysis

Liver and muscle glycogen levels were measured using the amyloglucosidase method [38] on tissues sampled 8 h after treatment (*n* = 6/line). Plasma glucose (Glucose RTU, bioMérieux, Marcy l’Etoile, France), triglycerides (PAP 150, bioMérieux) and free fatty acids (NEFA C kit, Wako Chemicals, Neuss, Germany) levels were determined in all samples using commercial kits adapted to a microplate format, according to the manufacturer’s recommendations.

### Western blot analysis

Expression of selected proteins was analyzed in the liver and muscle of fish (*n* = 6) sampled 3 h after treatment of glucose or saline solution. Frozen samples of liver (200 mg) and muscle (300 mg) were homogenized in 2 ml of buffer containing 150 mM NaCl, 10 mM Tris, 1 mM EGTA, 1 mM EDTA (pH 7.4), 100 mM sodium fluoride, 4 mM sodium pyrophosphate, 2 mM sodium orthovanadate, 1% Triton X-100, 0.5% NP-40-IGEPAL and 1.02 mg/ml protease inhibitor cocktail (Roche, Basel, Switzerland), using an Ultraturrax homogenizer. Tubes were kept in ice during the whole process to avoid protein denaturation. Homogenates were centrifuged at 1000 *g* for 15 min at 4°C and supernatants were again centrifuged at 20,000 *g* for 30 min. The resulting supernatants were recovered and stored at −80°C. The concentration of protein in each sample was determined using Bio-Rad protein assay kit (Bio-Rad Laboratories, Munich, Germany) with bovine serum albumin as standard. Liver and muscle protein lysates (10 µg of protein for Akt; 20 µg for AMPK) were subjected to SDS-PAGE and Western blotting using appropriate antibodies. Anti-phospho Akt (Ser^473^), anti-carboxyl terminal Akt, anti-phospho AMPK (Thr^172^) and anti-AMPK antibodies were used (Cell signaling Technology, Saint Quentin Yvelings, France). All these antibodies have been shown to cross-react successfully with rainbow trout proteins of interest [16], [33]. After washing, membranes were incubated with an IRDye infrared secondary antibody (LI-COR Biosciences) and spots were quantified by Odyssey Infrared Imaging System software (Version 3.0, LI-COR Biosciences).

### Gene expression analysis

Analyses of mRNA levels were performed on samples from the liver and white skeletal muscle collected at 3, 8 and 12 h after treatment with glucose or saline solution (n = 6 per each tissue, treatment and post-injection time). Total RNA was extracted by using Trizol reagent (Invitrogen, Carlsbad, CA, USA) according to the manufacturer’s recommendations. One microgram of the resulting total RNA was reverse transcribed into cDNA using the SuperScript III RNaseH-reverse transcriptase kit (Invitrogen) and random primers (Promega, Charbonnières, France) according to the instructions of each manufacturer, including no RNA and no reverse transcriptase control. Target gene expression levels were determined by quantitative real-time RT-PCR, using specific primers [32], [33], [39], [40].

Quantitative RT-PCR was carried out on a LightCycle 480 II (Roche Diagnostics, Neuilly-sur-Seine, France) using SYBR Green I Master (Roche Diagnostics GmbH, Mannheim, Germany). The transcripts assessed were glucokinase (GK), 6-phosphofructo-1-kinase (6PFK) and pyruvate kinase (PK) for glycolysis; glucose-6-phosphatase (G6Pase1 and G6Pase2), fructose 1,6-bisphosphatase (FBPase) and phosphoenolpyruvate carboxykinase (PEPCK) for gluconeogenesis; glucose facilitative transporter type 2 (GLUT2) and 4 (GLUT4) for glucose transport; sterol regulatory element binding protein 1c (SREBP1c) for transcription factor regulating lipogenesis; glucose 6-phosphate dehydrogenase (G6PD), ATP citrate lyase (ACLY), acetyl-CoA carboxylase (ACC) and fatty acid synthase (FAS) for *de novo* lipogenesis; carnitine palmitoyl transferase 1 (CPT1a and CPT1b), 3-hydroxyacyl-CoA dehydrogenase (HOAD) and acyl-CoA oxidase (ACO) for fatty acid β-oxidation. No template control was applied for each primer. 18S and β-actin were used as non-regulated reference genes in liver and muscle respectively where their expression was stable. Relative quantification of target gene expression was performed using the mathematical model described by Pfaffl [41].

### Statistical analysis

Results are mostly expressed as means ± SD. *t*-test was used to assess the significant difference in muscle fat content (*n* = 5) between lines. Results from plasma metabolite levels (*n* = 8) were analyzed using two-way ANOVA to assess the differences between lines, sampling time and interaction. For glycogen content (n = 6), protein phosphorylation levels and gene expression (*n* = 6) the effect of treatment, line and treatment × line interaction were tested by two-way ANOVA. When an interaction was significant, means were compared using the Student-Newman-Keuls multiple comparison test. For all statistical analyses, the level of significance was considered at *P*<0.05.

## Results

### Plasma metabolites levels

Plasma concentration of specific metabolites at 3, 8 and 12 h after intraperitoneal injection of glucose or saline solution are presented in Table 1. In both lines, glucose administration resulted in a pronounced and persistent hyperglycemia, as observed at 3 h post-injection. Thereafter at 8 h post-injection, glycemia had returned to basal values. However, L line exhibited hyperglycemia again at 12 h after glucose treatment, in contrast to the normal glycemia observed in F line. Plasma triglycerides followed a time-response pattern similar to glycemia profile in L line at 12 h post-glucose treatment. In the saline injected fish, F line was found to have higher plasma triglyceride and free fatty acid levels than L line, but no such genotypic difference was observed in the glucose treated fish.

**Table 1:** Plasma metabolite levels in L line and F line after intraperitoneal (IP) administration of glucose (250 mg/kg) or saline solution.

Plasma metabolite	Treatment	L line			F line			*P* value		
		3 h	8 h	12 h	3 h	8 h	12 h	Time	Line	Time×Line
Glucose (mmol/L)	Saline	5.20±1.18^b^	6.02±2.30	3.67±0.43^b^	5.38±2.46^b^	4.74±1.39	4.37±1.21	0.04	0.79	0.24
	Glucose	9.61±2.35^a^	5.70±1.81	9.03±4.30^a^	8.55±3.64^a^	4.69±1.05	5.58±2.38	0.001	0.03	0.38
Triglycerides (mmol/L)	Saline	1.31±0.64	1.10±0.42^a^	0.72±0.39^b^	1.58±0.69	1.99±0.45^a^	1.48±0.64^a^	0.11	0.003	0.07
	Glucose	1.66±0.75	0.61±0.36^b^	2.26±1.42^a^	2.01±1.18	0.89±0.61^b^	0.86±0.40^b^	<10^−3^	0.34	0.01
Free fatty acids (mEq/L)	Saline	0.17±0.04	0.14±0.05	0.26±0.10	0.26±0.15	0.27±0.11^a^	0.47±0.33	0.01	0.004	0.59
	Glucose	0.15±0.07	0.12±0.05	0.18±0.06	0.21±0.14	0.17±0.06^b^	0.27±0.05	0.99	0.99	0.37

Data are presented as means ± SD (*n* = 8). The effect of sampling time, line and interaction were analyzed by two-way ANOVA for each treatment group (*P*<0.05). At each sampling time, significant differences between glucose and saline treatment are represented with different superscripts (a, b) (*t*-test, *P*<0.05).

### Tissue glycogen content

**Figure 1:**
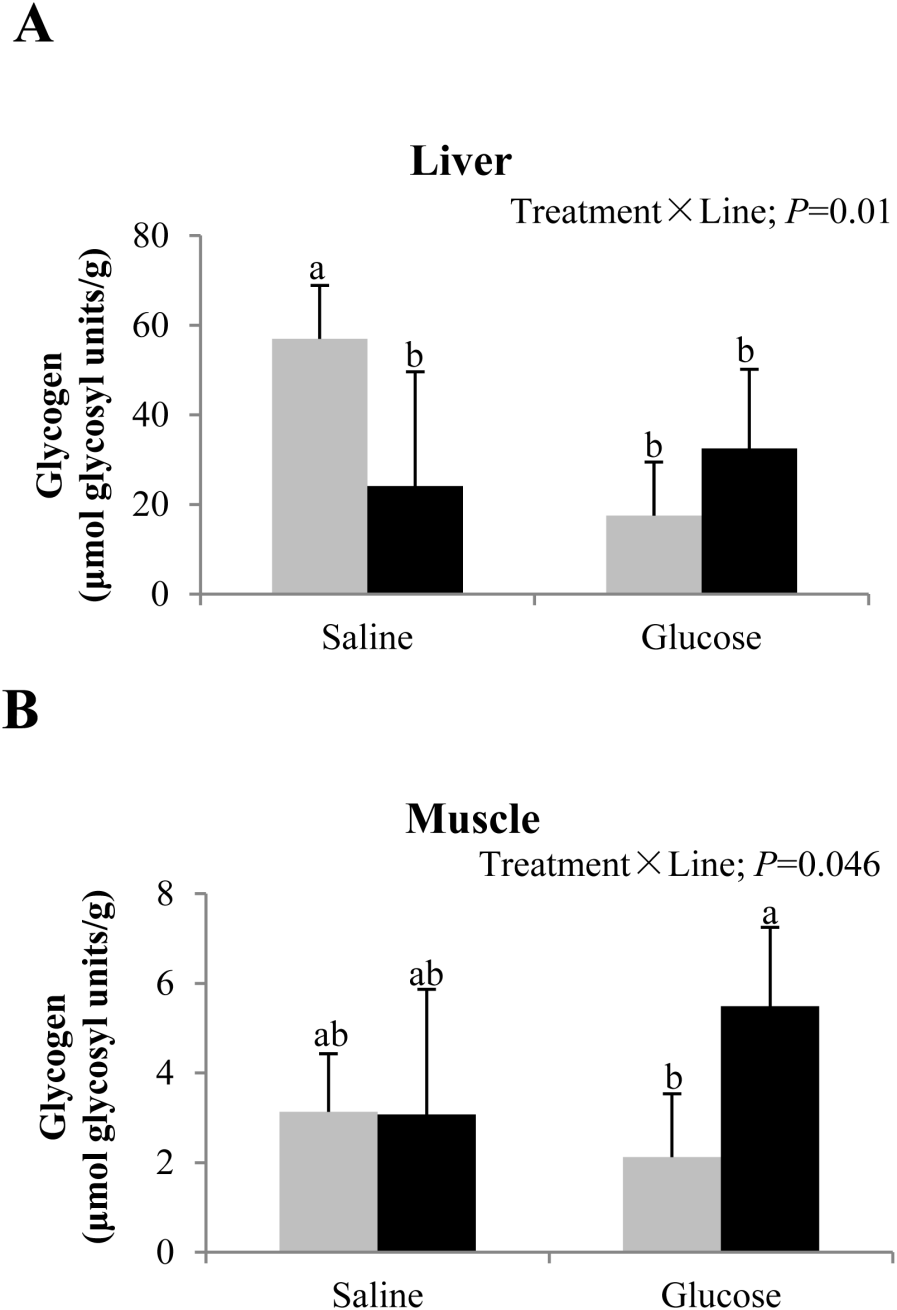
Glycogen content in liver (A) and muscle (B) of rainbow trout from L line (L; grey bars) and F line (F; black bars), 3 h after IP administration of glucose (Glu) or saline (Sal) solution. Data are presented as means ± SD (*n* = 6) and were analyzed using two-way ANOVA (*P*<0.05). In case of interaction, mean values not sharing a common lowercase letter are significantly different from each other.

Regarding tissue glycogen content (Fig. 1), a significant treatment × line interaction was observed in both liver and muscle. Glucose treatment was linked to liver glycogen depletion in L line, whereas in F line, it was associated with unaltered liver glycogen content in F line. With regard to glycogen content in muscle, a slight but not significant decrease was found in L line, whereas an inverse effect was observed in F line.

### Insulin signaling pathway and cellular energy sensor (AMPK)

**Figure 2:**
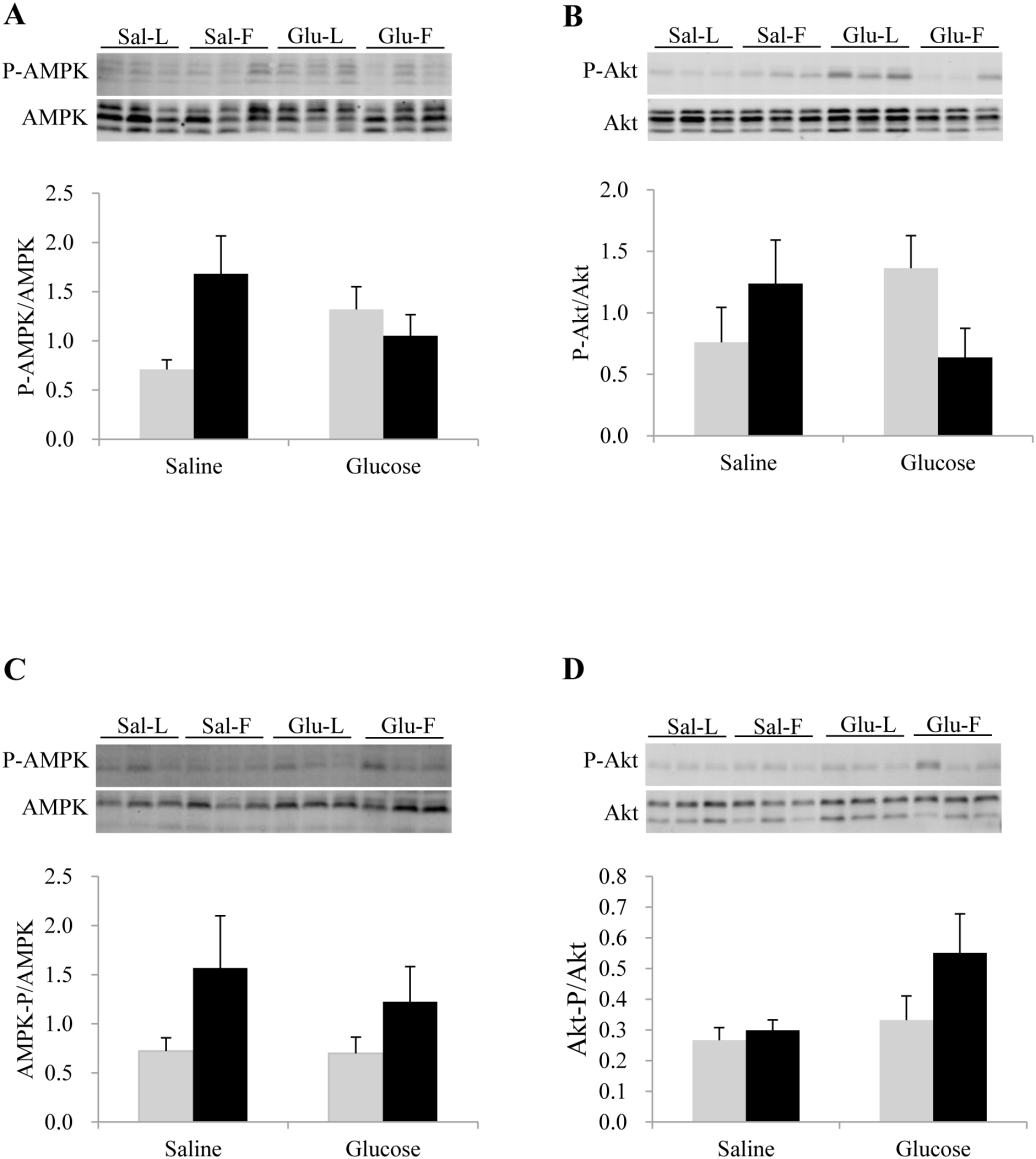
Western blot analysis of AMPK and Akt phosphorylation in rainbow trout liver (A) and muscle (B) samples from L line (L; grey bars) and F line (F; black bars), 3 h after IP administration of glucose or saline solution. 10 and 20 µg of total protein per lane for Akt and AMPK were loaded on the gel respectively. Western blots were performed on 6 individual samples per treatment and a representative blot is shown here. Data were analyzed using two-way ANOVA (P<0.05), followed by a Student-Newman-Keuls multiple comparison test.

Changes in the phosphorylation status of components of the insulin signaling pathway (Akt-TOR) and major cellular energy sensor (AMPK) were analyzed in the liver and muscle of the two trout lines sampled 3 h after treatment, using Western blot. No significant difference was noted in the ratio of phosphorylated to total protein of Akt and AMPK between the two genotypes as well as treatment, in both liver and muscle (Fig. 2).

### Messenger RNA levels of target genes

**Figure 3:**
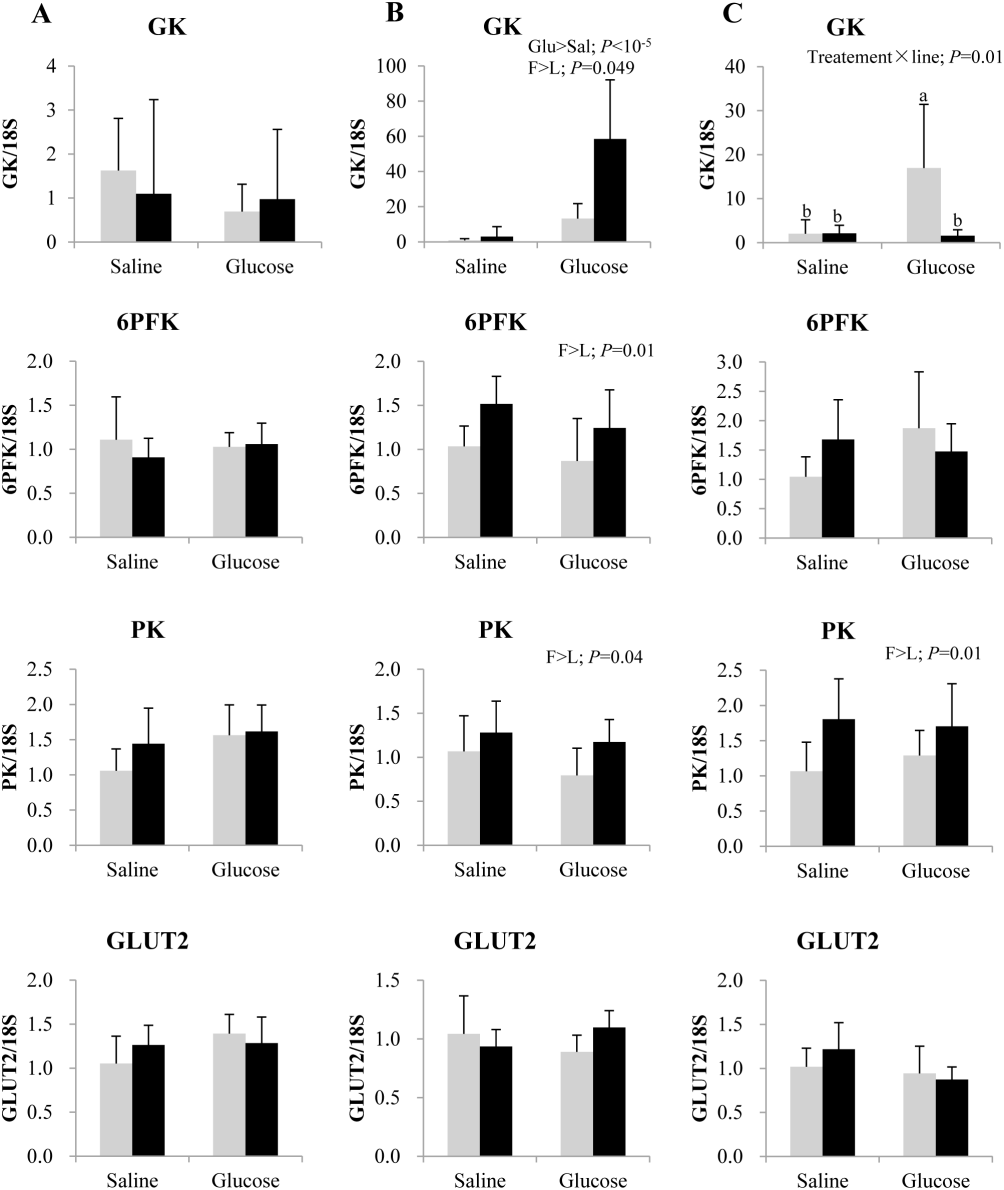
Gene expression of selected glycolytic enzymes and glucose transporter in the liver of rainbow trout from L line (L; grey bars) and F line (F; black bars) at 3 h (A), 8 h (B) and 12 h (C) after IP administration of glucose or saline solution. Glucokinase (GK), 6-phosphofructo-1-kinase (6PFK), pyruvate kinase (PK) and glucose transporter type 2 (GLUT2) mRNA levels were measured using real-time quantitative RT-PCR. Expression values were normalized by 18S-ribosomal RNA transcripts. Results are expressed as means ± SD (*n* = 6) and were analyzed using two-way ANOVA (*P*<0.05), followed by a Student-Newman-Keuls multiple comparison test. In case of interaction, mean values not sharing a common lowercase letter are significantly different from each other.

Relative fold difference in the mRNA levels of hepatic glycolytic enzymes at 3, 8 and 12 h post-injection is shown in Fig. 3. GK mRNA level was markedly induced in both lines at 8 h after glucose treatment (*P*<10^−5^); this induction was more pronounced in the F line. Compared to L line, the transcripts of 6PFK and PK were also more abundant in the F line at 8 h post-injection, irrespective of saline or glucose treatment. At 12 h post-injection, a significant interaction between treatment and line was observed for GK expression (*P* = 0.01), where glucose administration up-regulated GK expression only in L line. Hepatic PK expression however remained enhanced in F line at 12 h. GLUT2 mRNA level in the liver was not modified by glucose treatment and did not vary between the two genotypes.

**Figure 4:**
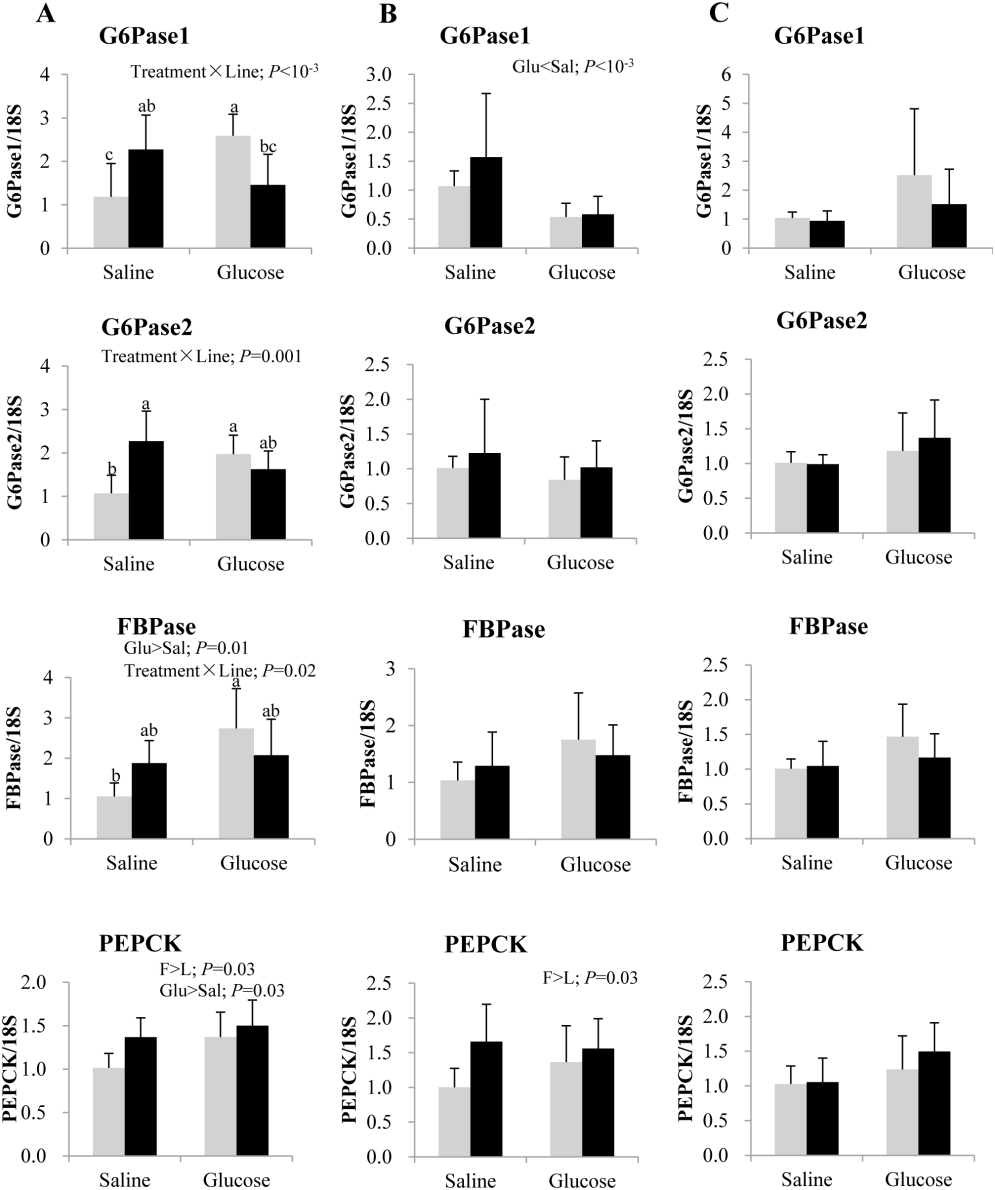
Gene expression of selected gluconeogenic enzymes in the liver of rainbow trout from L line (L; grey bars) and F line (F; black bars) at 3 h (A), 8 h (B) and 12 h (C) after IP administration of glucose or saline solution. Glucose-6-phosphatase isoform 1 (G6Pase1) and isoform 2 (G6Pase2), fructose 1,6-bisphosphatase (FBPase) and phosphoenolpyruvate carboxykinase (PEPCK) mRNA levels were measured using real-time quantitative RT-PCR. Expression values were normalized by 18S-ribosomal RNA transcripts. Results are expressed as means ± SD (*n* = 6) and were analyzed using two-way ANOVA (*P*<0.05), followed by a Student-Newman-Keuls multiple comparison test. In case of interaction, mean values not sharing a common lowercase letter are significantly different from each other.

Relative transcript levels of key gluconeogenic enzymes in the liver are presented in Fig. 4. In both lines, glucose treatment was associated with a decrease in the expression of G6Pase1 at 8 h post-injection, but conversely it was found to enhance the expression of FBPase and PEPCK at 3 h post-injection. Particularly, PEPCK transcripts were more abundant in F line than L line, irrespective of treatment. In addition, disordinal interaction between treatment and line was observed for G6Pase1, G6Pase2 and FBPase mRNA levels at 3 h post-injection, where glucose loading up-regulated the expression of these enzymes specifically in the L line.

**Figure 5:**
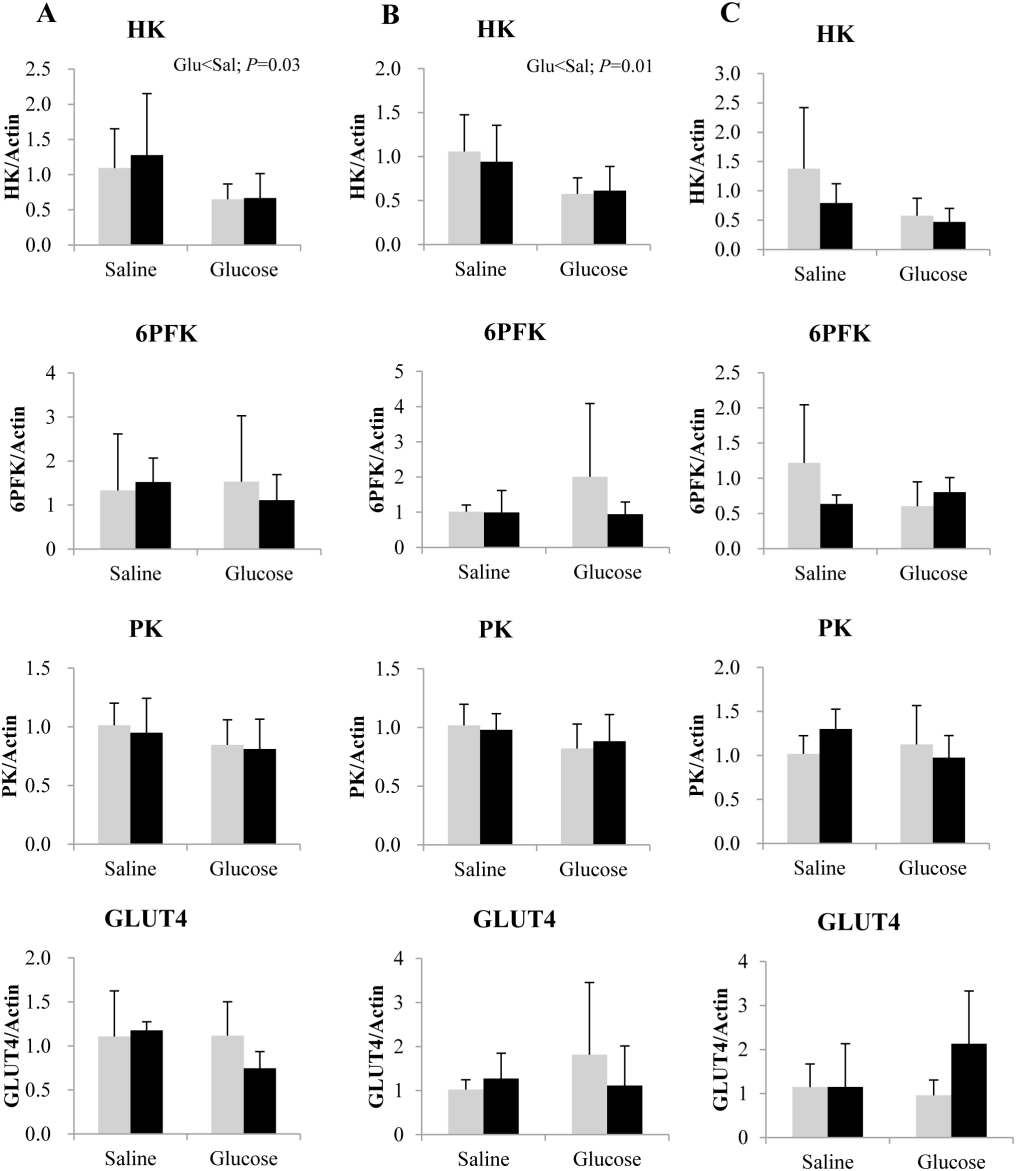
Gene expression of selected glycolytic enzymes and glucose transporter in the muscle of rainbow trout from L line (L; grey bars) and F line (F; black bars) at 3 h (A), 8 h (B) and 12 h (C) after IP administration of glucose or saline solution. Hexokinase (HK), 6-phosphofructo-1-kinase (6PFK), pyruvate kinase (PK) and glucose transporter type 4 (GLUT4) mRNA levels were measured using real-time quantitative RT-PCR. Expression values were normalized by β-actin-transcripts. Results are expressed as means ± SD (*n* = 6) and were analyzed using two-way ANOVA (*P*<0.05), followed by a Student-Newman-Keuls multiple comparison test.

Concerning the expression of glycolytic markers in white muscle (Fig. 5), we observed a reduction in the mRNA level of HK in both lines, at 3 and 8 h after glucose administration. Apart from that, no other changes were found in the transcript levels of HK, 6PFK, PK and GLUT4 due to treatment or genotype.

**Figure 6:**
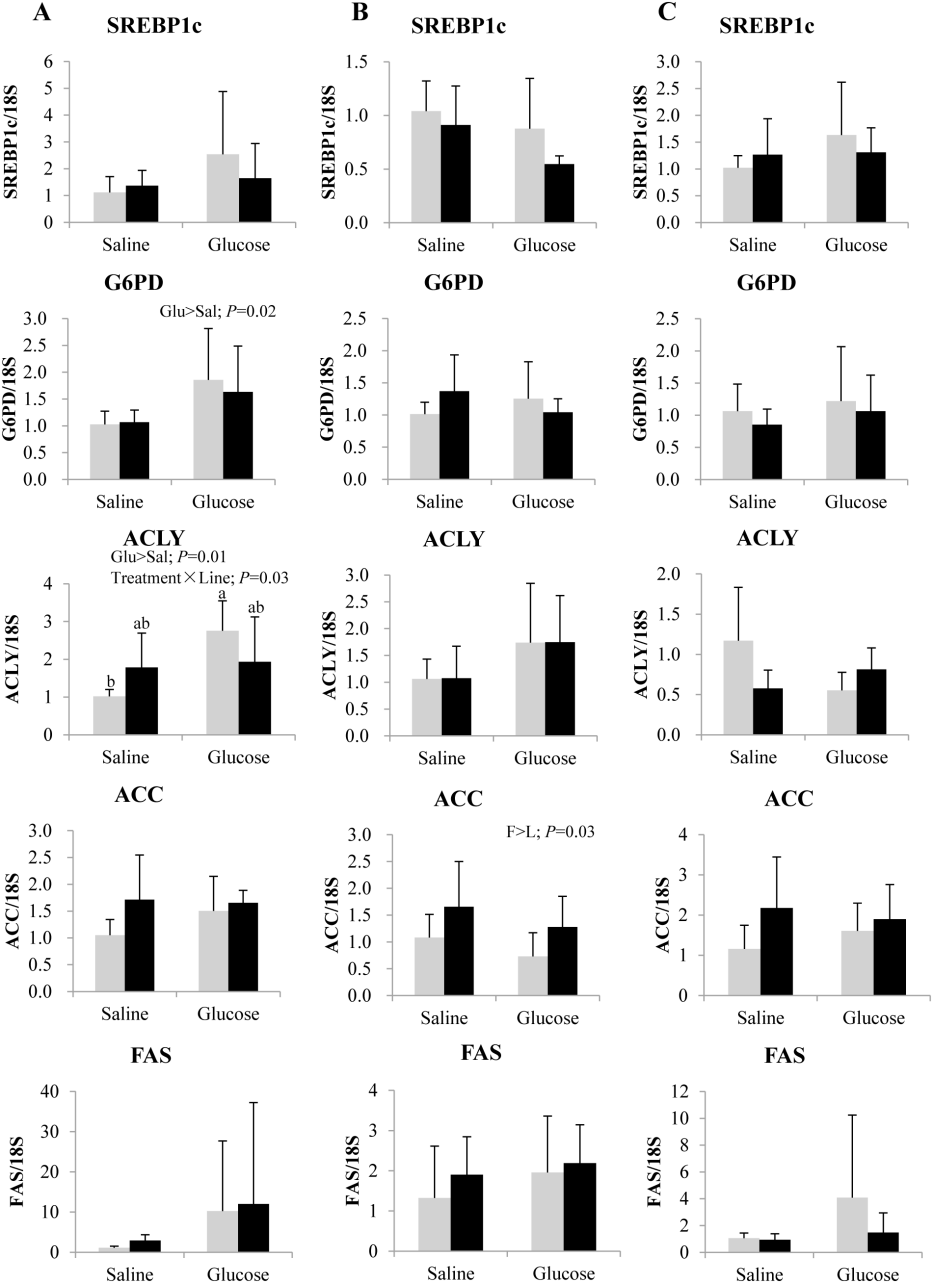
Gene expression of selected transcription factors and enzymes involved in NADPH generation and lipogenesis in the liver of rainbow trout from L line (L; grey bars) and F line (F; black bars) at 3 h (A), 8 h (B) and 12 h (C) after IP administration of glucose or saline solution. Sterol regulatory element binding protein 1c (SREBP1c), glucose 6-phosphate dehydrogenase (G6PD), ATP citrate lyase (ACLY), acetyl-CoA carboxylase (ACC) and fatty acid synthase (FAS) mRNA levels were measured using real-time quantitative RT-PCR. Expression values were normalized by 18S-ribosomal RNA transcripts. Results are expressed as means ± SD (*n* = 6) and were analyzed using two-way ANOVA (*P*<0.05), followed by a Student-Newman-Keuls multiple comparison test. In case of interaction, mean values not sharing a common lowercase letter are significantly different from each other.

**Figure 7:**
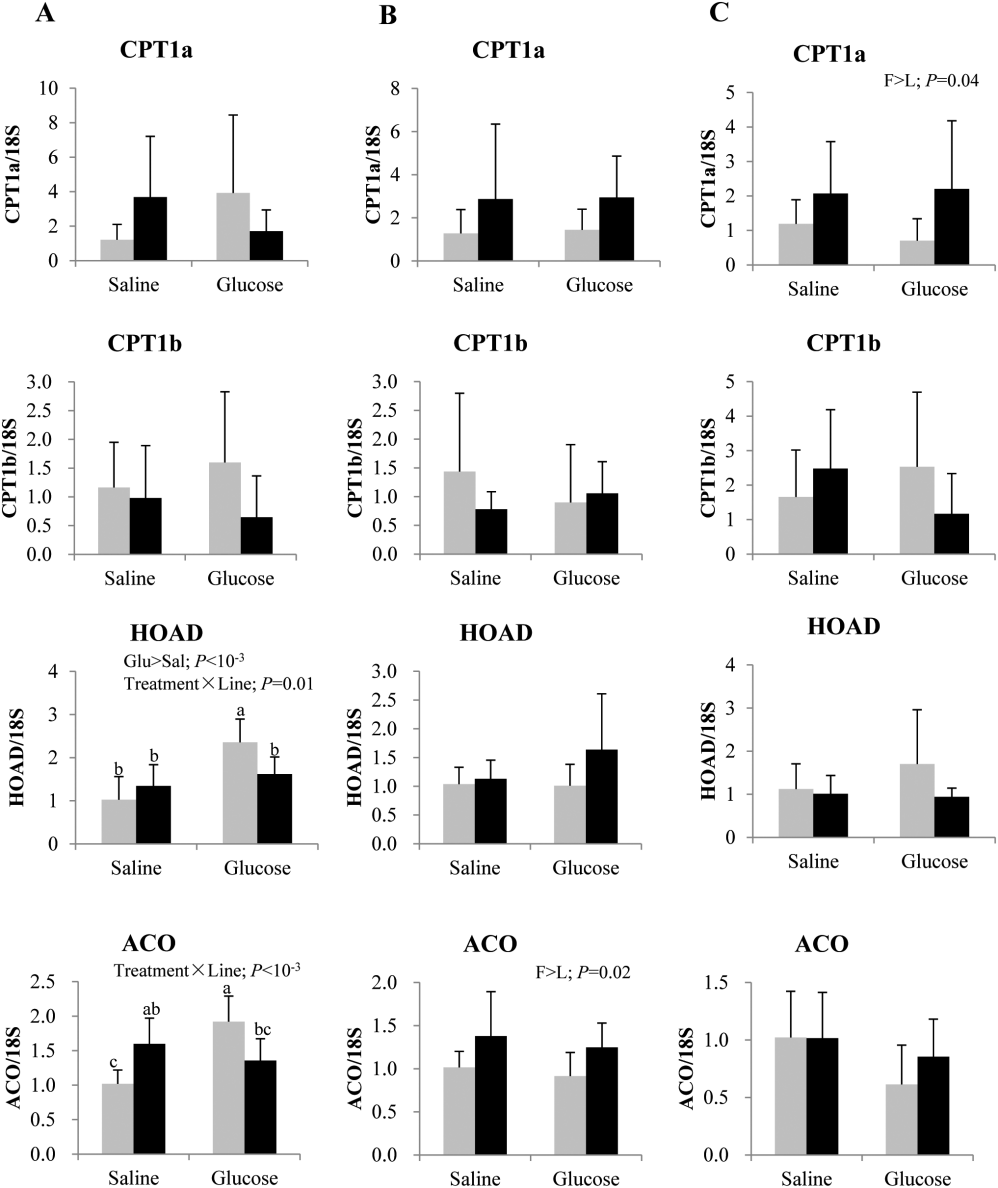
Gene expression of selected fatty acid oxidation enzymes in the liver of rainbow trout from L line (L; grey bars) and F line (F; black bars) at 3 h (A), 8 h (B) and 12 h (C) after IP administration of glucose or saline solution. Carnitine palmitoyl transferase isoform 1 a (CPT1a) and b (CPT1b), 3-hydroxyacyl-CoA dehydrogenase (HOAD) and acyl-CoA oxidase (ACO) mRNA levels were measured using real-time quantitative RT-PCR. Expression values were normalized by 18S-ribosomal RNA transcripts. Results are expressed as means ± SD (*n* = 6) and were analyzed using two-way ANOVA (*P*<0.05), followed by a Student-Newman-Keuls multiple comparison test. Mean values not sharing a common lowercase letter are significantly different from each other.

As represented in Fig. 6, at 3 h post-injection, mRNA levels of the NADPH generating pentose phosphate pathway enzyme G6PD was enhanced by glucose administration in both lines. Similar induction was observed also for ACLY expression, but only in the L line, leading to a significant interaction. Transcripts of SREBP1c, ACC and FAS were not significantly influenced by the glucose load. Except for an enhanced expression of ACC in the F line at 8 h post-injection, no genotypic difference was noted in the target lipogenic markers. Concerning fatty acid β-oxidation in the liver (Fig. 7), the F line displayed higher mRNA levels of ACO and CPT1a than the L line at 8 and 12 h post-injection, respectively, regardless of the treatment. Disordinal treatment × line interaction was observed in HOAD and ACO expression at 3 h post treatment, with a significant induction in the L line after a glucose load.

**Figure 8:**
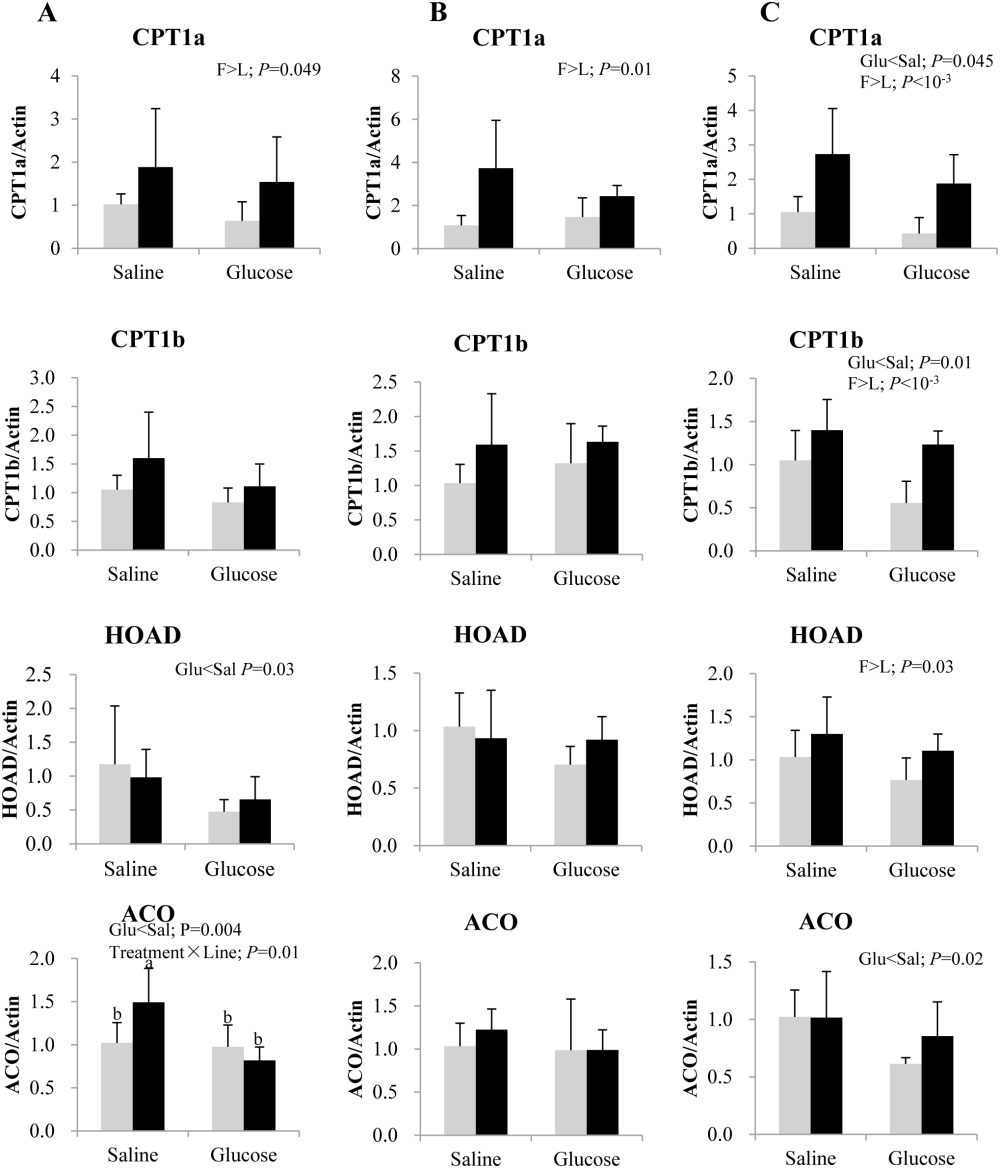
Gene expression of selected fatty acid oxidation enzymes in the muscle of rainbow trout from L line (L; grey bars) and F line (F; black bars) at 3 h (A), 8 h (B) and 12 h (C) after IP administration of glucose or saline solution. Carnitine palmitoyl transferase isoform 1 a (CPT1a) and b (CPT1b), 3-hydroxyacyl-CoA dehydrogenase (HOAD) and acyl-CoA oxidase (ACO) mRNA levels were measured using real-time quantitative RT-PCR. Expression values were normalized by β-actin-transcripts. Results are expressed as means ± SD (*n* = 6) and were analyzed using two-way ANOVA (*P*<0.05), followed by a Student-Newman-Keuls multiple comparison test. In case of interaction, mean values not sharing a common lowercase letter are significantly different from each other.

Changes in relative mRNA levels of key enzymes involved in muscle fatty acid oxidation are shown in Fig. 8. Glucose administration was found to down-regulate the expression of CPT1 isoforms, HOAD and ACO in both lines, at 3 or 12 h post-injection, irrespective of the treatment. F line exhibited consistently higher CPT1a expression than L line at all the post-injection time points. Similar genotypic difference was observed also in the transcript levels of CPT1b and HOAD at 12 h post-injection. Finally, a significant treatment × line interaction was observed in ACO expression at 3 h post-injection, where glucose loading reduced ACO expression specifically in F line.

## Discussion

Previous nutritional studies in the two selected trout lines suggested that the F line displayed a higher ability to metabolize glucose and to synthesize lipids *de novo* than the L line [16], [32], [33]. We thus postulated that the F line has a better capability to maintain glucose homeostasis after a dietary glucose load than L line. To check this hypothesis, the two rainbow trout lines were intraperitoneally injected with glucose or saline solution to investigate the genotypic differences in the regulation of glucose and lipid metabolism after a high glucose flux. This study is clearly different from our previous study based on the use of dietary carbohydrates [16] related to the route of the glucose supplementation (intake of dietary carbohydrates [16] versus intraperitoneal glucose injection) and the nutritional status of the fish (fed fish [16] versus unfed fish). Moreover, the present study describes the potential differential regulation of metabolism by glucose between two rainbow trout lines which is highly interesting for a better understanding of the glucose use in carnivorous animals. Indeed, we focused our analysis on the Akt/TOR signaling pathway and the expression of several target genes related to glucose and lipid metabolism. The time-dependent regulation of metabolic gene expression by glucose is different for each gene: some are differentially expressed already 3 h after injection (the hepatic gluconeogenic genes for example) and others are differently expressed only 12 h after injection (fatty acid oxidation genes in muscle for example) suggesting that the genes are not regulated by glucose with similar molecular mechanisms.

### Glucose induces glucokinase and does not regulate gluconeogenesis at a molecular level in the liver of both trout lines

As expected, a strong and persistent hyperglycemia was induced after glucose treatment in both rainbow trout lines, confirming the poor ability of this species to regulate glucose homeostasis [19], [42]. The high glycemia had no effect on the mRNA levels of hepatic glucose transporter GLUT2, probably due to the fact that this gene is present constitutively in the plasma membrane [43]. Moreover, the expression of GLUT2 has been observed to be high in rainbow trout, irrespective of the nutritional status [44]. Concerning hepatic glycolysis, Borrebaek et al. reported that high starch content in the diet up-regulated GK activity in Atlantic salmon [45]. Similarly in rainbow trout, a strong induction of GK expression was observed following the intake of a carbohydrate rich diet [42]. In agreement with these demonstrations, we found that the mRNA level of GK was markedly induced by glucose treatment in both trout lines, even though there was no significant increase of insulin signaling as indicated by lack of activation of Akt/TOR pathway in the present study.

With respect to gluconeogenesis, glucose was found to enhance the transcript levels of FBPase and PEPCK in the liver of both trout lines, reinforcing the fact that in carnivorous fish, glucose does not exert a mammalian like inhibitory effect on the gluconeogenic pathway [16], [44], [46], [47]. In particular, the augmentation of the gluconeogenic potential that we observed after a glucose load in these two trout lines, have also been described in other carnivorous fish such as red sea bream and yellowtail [14]. Nevertheless, in concordance with the lowered glycemic values at 8 h after glucose treatment, hepatic G6Pase was found to be down-regulated in both lines.

In the white skeletal muscle of the glucose treated fish from both lines, we observed a paradoxical decrease in the expression of HK, which indicates an atypical regulation of muscle glucose metabolism in response to high influx of glucose, corroborating previous finding in salmonids [16], [19]. This observation also serves as an evidence for the poor capacity of glucose utilization in the peripheral tissues of carnivorous fish [1], irrespective of the genotype.

### F line exhibits an enhanced hepatic glycolytic and gluconeogenic potential, as compared to L line

Regarding the genotype effect, F line apparently had a more efficient and stable glucose clearance mechanism to deal with a glucose load than L line, in agreement with previous findings that reported significantly lower levels of glucose in the F line at 24 h after refeeding [33]. Moreover, the return of plasma glucose level to basal values at 8 h after glucose treatment in F line was relatively much faster than the 24 h glycemia recovery period previously reported in rainbow trout, after administering a similar dose of glucose [7]. These differences in glycemia profiles between the two lines after glucose challenge could be due to genetic difference as a side effect of the selection for the target trait, i.e. the muscle fat content. Even if the existence of a genetic component that would determine the ability to utilize glucose is unknown in fish, it can be hypothesized, as suggested by the variation in glucose tolerance among three natural strains of Chinook salmon [30] and by selectively bred fast growing families of rainbow trout which have higher plasma insulin levels than their slow growing counterparts [48]. The higher efficiency of plasma glucose clearance in F line was basically associated with its enhanced hepatic glycolytic potential (higher transcript levels of GK, 6PFK and PK), consistent with previous observations [16], [32]. Overall, these data suggest that genetic selection could be a determinant of glucose homeostasis as in Chinook salmon [30]. However, it is important to note that, the transcript levels of gluconeogenic enzyme PEPCK were also higher in F line, suggesting a greater endogenous glucose synthesis potential than in L line. Concomitant weaker control of gluconeogenesis and enhanced glycolytic potential was similarly observed in the 4th generation F line fish, irrespective of dietary carbohydrate levels [16].

In fish, liver is the major tissue that regulates glucose disposal [49] and generally, hepatic glycogen content is higher in fish fed a carbohydrate-rich diet than in those fed a carbohydrate-free diet due to excess glucose storage [4], [50]. However on a per fish basis, muscle is more important for glucose disposal because it represents 50% of the total body mass [19], [51]. With respect to tissue glycogen deposition in both lines, we observed a significant interaction between glucose treatment and genotype in both liver and muscle. The hyperglycemia found in L line at 12 h after glucose treatment can be probably explained by an apparent lower glycolytic potential in the liver of this line compared to the F line concomitantly with a decreased potential of glycogen synthesis in liver at 8 h post-injection. This in turn may be related to the lower muscle lipid content, i.e., when the availability of fatty acids is limited, glucose serves as the source of oxidative fuel in this line. On the other hand, normoglycemia was observed in F line at 12 h post-injection, in concordance with a slight but not statistically significant increase in muscle glycogen deposition. These cumulative findings endorse our hypothesis that F line has a better ability to maintain glucose homeostasis than L line.

### Glucose induces hepatic G6PD and decreases the expression of fatty acid oxidation markers in the muscle of both rainbow trout lines

Lipogenesis possibly plays a crucial role in the glucose homeostasis of rainbow trout, by converting excess glucose into lipids [4]. G6PD and ACLY are the key enzymes that provide NADPH for fatty acid biosynthesis and divert glycolytic carbon flux into lipid biosynthesis, respectively. We found that glucose administration enhanced the transcript levels of G6PD and ACLY, but not those of FAS and SREBP1c, similar to the observation made after long term feeding of a carbohydrate rich diet in the 4th generation fish [16]. The absence of induction of SREBP1c and FAS expression after glucose treatment in the present study might be partly related to the 48 h-fasting applied before injection.

In mammals, glucose uptake inhibits fatty acid oxidation, both in mitochondria and peroxisome due to insulin secretion and action [52], [53]. However in these two trout lines, insulin signaling was probably not significantly induced by glucose administration, as reflected by the unaltered phosphorylation status of the components of Akt/TOR signaling pathway in the liver and muscle at 3 h post-injection, in contrast to our previous study [16]. This discrepancy may be caused by the experimental differences between the two studies: intake of dietary carbohydrates [16] may be able to induce insulin secretion related to increase of blood glucose plus other incretin factors (glucose-dependent insulinotropic polypeptide (GIP) and glucagon-like peptide 1 (GLP-1) for example) [54] which are not present after glucose injection. Furthermore, glucose is known to stimulate hepatic lipolysis in rainbow trout, both *in vivo* and *in vitro*, by enhancing the activity of lipolytic enzymes [55], [56]. Somatostatin was found to mediate the stimulation of hepatic lipolysis in several species such as rainbow trout, coho salmon and lamprey [57], [58]. Hence, the glucose enhanced HOAD mRNA levels that we observed, might be resulting from elevated plasma somatostatin concentration.

The effect of glucose treatment on muscle fatty acid oxidation was different from our observations in the liver, confirming tissue specific regulation [59]. Expression of the genes encoding the fatty acid oxidation enzymes, i.e. CPT1a, CPT1b, HOAD and ACO were significantly inhibited by glucose in the muscle, differing from earlier reports of inertness in rainbow trout fed a high carbohydrate diet [16], [27], [59]. This inconsistency may be possibly due to the route of glucose administration/uptake (intraperitoneal injection of glucose *vs.* intake of a high carbohydrate diet) and the nutritional status of the fish (fasted *vs.* fed). Inhibition of fatty acid oxidation markers when coupled together with the decrease in the level of plasma triglycerides, suggests that the external glucose load might trigger a switch from the use of fatty acids as fuel in fasted fish to the use of glucose as fuel in glucose treated fish.

### F line exhibits a higher potential of fatty acid oxidation than L line, at the molecular level

In contrast to the higher lipogenic potential demonstrated in F line of the previous generation [16], [33], we found no genotypic differences in the target genes related to *de novo* lipogenesis, except for an elevated expression of ACC in the F line. Thus, the higher muscle fat content in F line cannot be strictly attributed to its lipogenic ability, as previously noted [32]. Likewise, increased muscle fat content in F line is also not due to a decrease in fatty acid oxidation capacity [32]. Conversely, we found an enhanced fatty acid oxidation potential (especially at the fatty acid transport level with CPT1) in the liver and muscle of F line, contrary to our previous observations [16], [32], [33]. Even though we cannot eliminate a putative difference linked to the generation of selection (5th *vs.* 3rd and 4th), these discrepancies could be due to the nutritional status of the fish, because this is the first time that we analyzed fasted fish (48 h prior to the peritoneal injection). Indeed, unfed fish use fatty acids as the preferential energy source [60] in contrast to fed fish; we can hypothesize that fatty acid potential could be strongly changed in this context in the fish lines.

Previous studies demonstrated that there were very few line × diet interactions for the markers of lipid metabolism in both 3rd and 4th generations of selection [16], [32]. In agreement with this, limited but significant interactions were observed between glucose treatment and genotype in the hepatic expression of ACLY, HOAD and ACO, where glucose specifically enhanced their expression in the L line. In particular, we can say that glucose affects hepatic fatty acid oxidation markers in a genotype specific manner.

## Conclusions

The present study mainly reveals the differences in the transcriptional regulation of glucose and lipid metabolism after a GTT between two genotypes of rainbow trout. In both lines, intraperitoneal administration of glucose led to a prolonged hyperglycemia, this relative inability to utilize high levels of glucose was associated with a lack of regulation of gluconeogenesis. However, compared to L line, regulation of glycemia was more stable in F line, linked to higher muscle glycogen content and an enhanced expression of glycolytic gene markers in the liver, after glucose treatment. Some of these observations are coherent with the findings of previous nutritional challenge studies [16], [32], [33] and support our hypothesis that the F line has a higher capability to maintain glucose homeostasis after a glucose load than the L line. Our data are also helpful for understanding glucose use in carnivorous fish. Therefore, genetic selection is apparently a possible way to modify the poor capability of glucose hemostasis in carnivorous fish, primarily by influencing the regulation of key enzymes involved in glucose and lipid metabolism.
